# Flavonoids and Their Metabolites: Prevention in Cardiovascular Diseases and Diabetes

**DOI:** 10.3390/diseases5030019

**Published:** 2017-09-05

**Authors:** Keti Zeka, Ketan Ruparelia, Randolph R. J. Arroo, Roberta Budriesi, Matteo Micucci

**Affiliations:** 1 Leicester School of Pharmacy, Faculty of Health and Life Sciences, De Montfort University, The Gateway, Leicester LE1 9BH, UK; kcruparel@dmu.ac.uk (K.R.); rrjarroo@dmu.ac.uk (R.R.J.A.); 2 Department of Pharmacy and Biotechnology, University of Bologna, Via Belmeloro 6, 40126 Bologna, Italy; roberta.budriesi@unibo.it (R.B.); matteo.micucci2@unibo.it (M.M.)

**Keywords:** antioxidants, atherosclerosis, diabetes, flavonoids, metabolites

## Abstract

The occurrence of atherosclerosis and diabetes is expanding rapidly worldwide. These two metabolic disorders often co-occur, and are part of what is often referred to as the metabolic syndrome. In order to determine future therapies, we propose that molecular mechanisms should be investigated. Once the aetiology of the metabolic syndrome is clear, a nutritional intervention should be assessed. Here we focus on the protective effects of some dietary flavonoids, and their metabolites. Further studies may also pave the way for development of novel drug candidates.

## 1. Atherosclerosis 

Atherosclerosis is known to predispose patients to myocardial infarction and stroke, and it is responsible for cardiovascular ailments that represent the main cause of death in industrialised societies. Disorders related to atherosclerosis, e.g., vascular inflammation and metabolic alterations, strongly favour the onset and progression of a range of chronic diseases. The onset of these pathological events is linked to several factors such as age, hypertension, diabetes mellitus, smoking, and dyslipidemia. 

Atherosclerosis is a chronic, inflammatory, fibro-proliferative disorder, primarily of the large- and medium-sized conduit arteries. The vascular production of reactive oxygen species (ROS), such as O_2_^−^, derived from NADPH oxidase (NOX)-catalysed oxidations, represents a key event on the road to endothelial dysfunction [1,2]. In fact, O_2_^−^ and H_2_O_2_ stimulate vascular smooth muscle cell (VSMC) hyperplasia and hypertrophy occurring along with intracellular alkalinization, increase of the intracellular free calcium concentration, activation of MAP kinase, and induction of proto-oncogene expression [3,4,5,6].

Endogenous and exogenous oxidants continuously trigger ROS generation in the vascular tissue which results in atherosclerosis, neointimal hyperplasia, and hypertension [7,8,9]. Different authors have reported that Angiotensin II-induced hypertension is, at least in part, due to oxidative stress [10,11]. 

Several natural extracts, e.g., from leaves of *Olea europea* L., or calyces of *Hibiscus sabdariffa* L. [12], exert multiple effects that result in an inhibition of atherosclerosis and hypertension. The effects include vasorelaxant, antioxidant and antinflammatory activities. Also, vegetal extracts rich in hydrolyzable tannins, such as *Castanea sativa* Mill. bark [13] and *Punica granatum* L. extracts [14] exert anti-atherogenic effects through several mechanisms including the suppression of inflammation and oxidative stress, the inhibition of adhesion molecules such as VCAM-1 and ICAM-1, and the lipid-lowering properties [15]. In addition, diets rich in flavonoids are consistently linked with beneficial effects in the primary prevention of cardiovascular events. 

**Figure 1:**
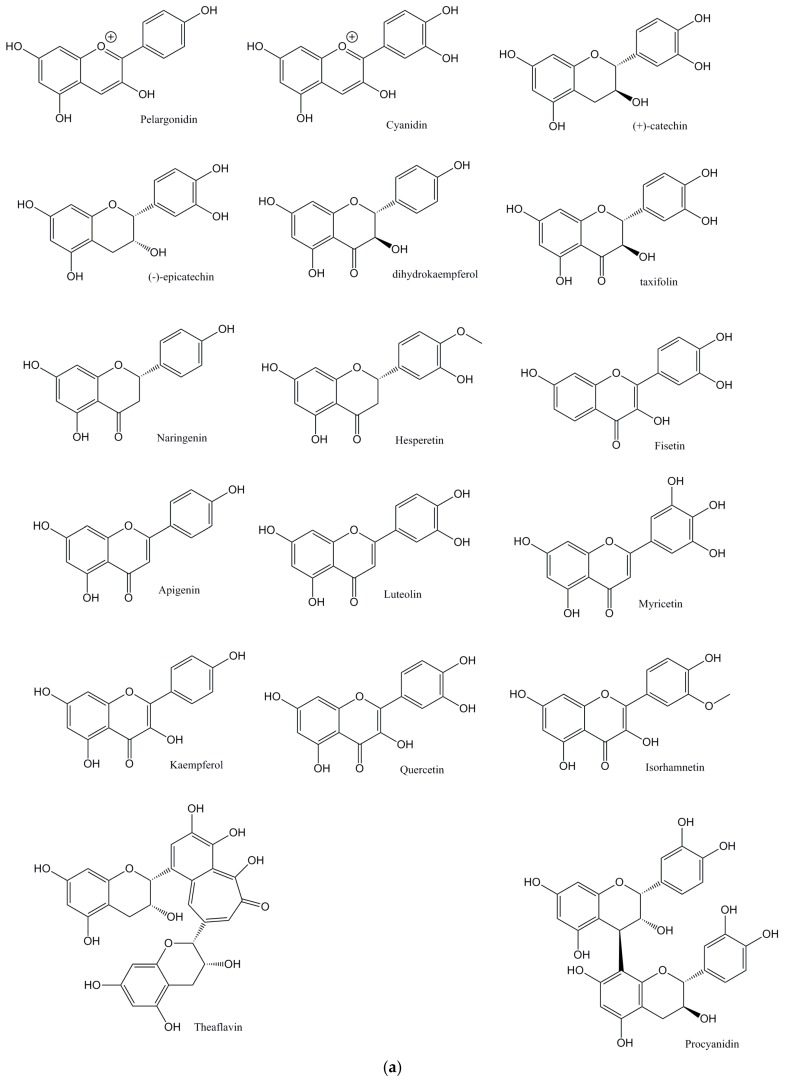

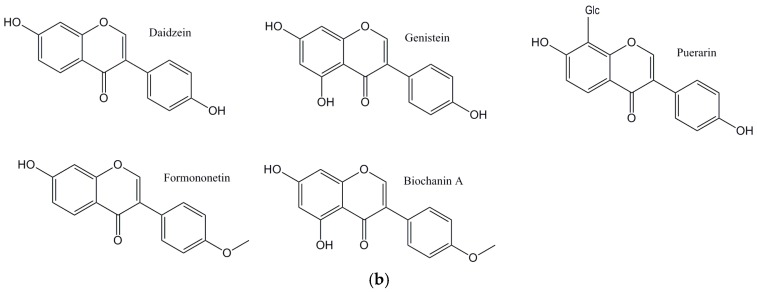
(**a**) Structures of the flavonoids discussed in this perspective; (**b**) Structures of the isoflavones discussed in this perspective.

The main aim of this perspective is to critically explore flavonoids (Figure 1a: Structures of the flavonoids discussed from this perspective; Figure 1b: Structures of the isoflavones discussed from this perspective) and their antioxidant activities, and their ability to affect lipid levels and the development of plaque, atherosclerosis, and its progression.

## 2. Flavonoids and Atherosclerosis

### 2.1. Direct Antioxidant Effects of Flavonoids

Dietary flavonoids commonly exert good antioxidant activity with the extent depending on the structure of the flavonoid [16]. It is generally accepted that the number and position of hydroxyl groups on B and A rings, and the extent of conjugation between the B and C rings are the main features affecting the flavonoids’ antioxidant activity [17].

**Figure 2:**
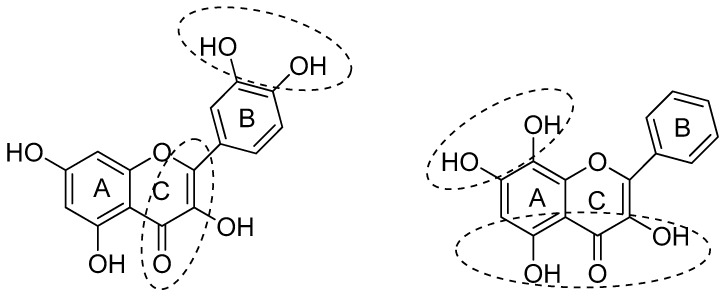
Structural features of flavonoids with high antioxidant activity, from Amić et al. [18].

The structural features of flavonoids that are necessary to exert radical scavenging and/or the antioxidative actions are described by the three criteria: (1) the *ortho*-dihydroxy (3′,4′-diOH, i.e., catechol) structure in the B ring, giving high stability to the flavonoid phenoxyl radicals via hydrogen bonding or by expanded electron delocalization; (2) the C2-C3 double bond (in conjugation with the 4-oxo group), which confers the co-planarity of the hetero-ring and contributes to radical stabilization via electron delocalization over all three ring systems; (3) the presence of both 3-OH and 5-OH (Figure 2) groups for the maximal radical scavenging capacity and the strongest radical absorption.

In addition, the lack of *o*-dihydroxy structure in the B ring can be compensated by hydroxyl substituents in a catechol structure on the A ring: this feature represents a larger determinant of flavonoid antiradical activity. The basic flavonoid structure is essential for the antioxidant activity only when a catechol configuration is absent. Glycosylation of flavonoids decreases their antioxidant activity. The block or the removal of the C3 OH group results in a reduction of antioxidative properties of flavonoids [17,18]. 

### 2.2. Indirect Antioxidant Effects of Flavonoids: The Involvment of ET-1 and NADPH Oxidase

Several experimental models of hypertension, atherosclerosis and diabetes are characterised by elevated levels of circulating ET-1 (Endothelin-1) [16,17,18,19].

ET-1 activates ETA/ETB receptors, leading to an augmented ROS production in vasculature, which results in endothelial dysfunction [20,21,22]. 

NADPH oxidase represents the principal source of intracellular ROS in vascular cells. It is a multi-subunit enzymatic complex that comprises two membrane-bound subunits named Nox (Nox-1, Nox-2 or gp91phox, Nox-4 or Nox-5) and p22phox, whose regulation occurs through cytoplasmic subunits like p47phox, p67phox and a low-molecular-weight G protein (rac 1 or rac 2) [23]. The signalling processes mediated by angiotensin II are multiphasic and occur at different times [24].

Following angiotensin II stimulation, activation of phospholipase C takes place within seconds and then catalyses an increase of free intracellular calcium resulting in vascular contraction, while the activation of signaling pathways which regulate protein synthesis and cell growth are delayed, and occur only after minutes or hours after angiotensin II stimulation [24,25]. Different experimental data suggest a direct correlation between angiotensin II stimulation and ROS generation, such as O_2_^−^, OH and H_2_O_2_ [26,27,28]. The ROS production caused by angiotensin II can be divided into an acute phase which comprises protein kinase C (PKC), c-Src, growth factor receptors transactivation and translocation of cytosolic p47phox to the membrane [29,30], and a sustained phase, in which the up-regulation of NADPH oxidase subunits (also involving PKC activation) occurs [31,32,33]. Interestingly, in several animal models of hypertension, long term administration of quercetin has been shown to restore the altered endothelial function [34]. This biological action may be due to the ability of quercetin to decrease the production of vascular O_2_^−^, occurring mainly through two mechanisms: (1) direct scavenging of O_2_^−^ and inhibition of O_2_^−^ generating enzyme [35]; (2) prevention of the expression of the genes involved in O_2_^−^ production, like NADPH oxidase subunits which can be induced by stimuli like angiotensin II [36] or endothelin-1 (ET-1) [37]. Three quercetin metabolites, i.e., quercetin-3-glucuronide, isorhamnetin-3-glucuronide and quercetin-3’-sulfate have been shown to prevent the impairment of endothelial-derived NO response while only quercetin and quercetin-3-glucuronide are able to prevent the in vitro ET-1 induced endothelial dysfunction. In particular, quercetin and its conjugated metabolites (though with a lower potency) are able to partially prevent the DECTA-induced alteration of contractile response of endothelium to Ach. Another activity of quercetin and its metabolites contributing to improve endothelial dysfunction, is the concentration-dependent inhibition of the production of O_2_^−^ dependent on NADPH-oxidase [38]. This effect may be due to the ability of quercetin and its metabolite isorhamnetin to inhibit protein kinase C (PKC). This enzyme is activated by ET-1 and is in turn involved in Nox activation. Furthermore, these flavonols also reduce ET-1-induced overexpression of p47phox, and thus provides further vascular protection. [39]. Also quercetin-3′-sulfate and quercetin-3-glucuronide (100 micromol/L) inhibit NADPH oxidase-derived. O_2_^−^ release [38] Given orally to spontaneously hypertensive rats, quercetin reduces the aortic expression of p47phox, and has not been detected in rats’ plasma, suggesting that the effect is mediated by quercetin metabolites [38]. These data are in agreement with in vivo experiments that have shown that long term treatment with quercetin reduces blood pressure and endothelial dysfunction in different experimental models for hypertension [39,40,41,42]. Other flavonoids able to inhibit NADPH oxidase are (−)-epicatechin [43,44,45,46], and its metabolites 3′-*O*-methyl-epicatechin, 4′-*O*-methyl epicatechin, epicatechin glucuronide (in particular the 3′ isomer, which is the main metabolite in human plasma) [47], and procyanidin B2 [48,49]. SAR studies have pointed out the fact that mono-*O*-methylation of catechol-type polyphenols change the biological activity from O_2_^−^ scavengers to exclusive NADPH oxidase inhibitors. In addition, the presence of a hydroxyl group in 4′ position of B-ring of flavonoids is an important feature for the inhibitory activity of flavonoids which is found in 3′-*O*-methylated (−)-epicatechin, (+)-catechin, quercetin, fisetin, luteolin, kaempferol, dihydrokaempferol, naringenin, able to exert this biological activity [50]. As regards the in vivo activity, the fact that the (−)-epicatechin action is significantly inhibited by low concentrations of 3,5-dinitrocatechol (DNC), an inhibitor of catechol-*O*-methyltransferase (COMT), suggests that the *O*-methylation of (−)-epicatechin is essential. Conversion of epicatechin to 3′ and 4′-methyl ethers was observed in vascular endothelial cells, and 3′-*O*-methyl epicatechin has been found in human plasma after oral intake. In vascular endothelial cells, epicatechin determines an increase of the steady-state level of nitric oxide, which is influenced by DNC. In contrast, the effect of the 3′-*O*-methyl epicatechin is not affected by DNC. The NO^−^ promoting action occurs through the inhibition of Nox, thus avoiding the O_2_^−^ generation. These observations made in mammalian cell cultures are in agreement with the outcome of different clinical studies which demonstrated that intake of high-flavanol cocoa or isolated epicatechin determines a transient improvement of endothelial function through elevated bioavailability of NO^−^ [50]. 

### 2.3. Indirect Antioxidant Effects of Flavonoids: The Involvement of Myeloperoxidase (MPO) and HOCl Scavange

Another pathway leading to an increase of oxidative stress is directed by myeloperoxidase (MPO), a secreted heme protein expressed at high levels in human atherosclerotic lesions, where it co-localizes in part with macrophages, which use hydrogen peroxide (H_2_O_2_) to execute oxidative reactions in the phagolysosome and extracellular milieu. This represents an important source of oxidative stress in the human artery wall [51]. The main end product of this pathway is HOCl, a strong chlorinating oxidant, able to chlorinate many different biomolecules, such as proteins, lipids, and nucleic acids at the site of inflammation [51,52,53]. In addition, HOCl oxidizes LDL and high-density lipoprotein in human arteries, resulting in the formation of atherogenic modified lipoproteins [51,54]. Some authors have suggested that the antiatherogenic effect of flavonoids may be due to MPO inhibition. In particular, quercetin and its metabolite quercetin-3-glucuronide have been shown to inhibit MPO in a concentration-dependent manner. In addition, plasma of quercetin-fed rats inhibits MPO, suggesting the possibility that quercetin and its metabolites may inhibit the MPO-derived oxidation reactions in vivo. Other flavonoids that inhibit MPO are, in order of potency, quercetin > kaempferol > fisetin > luteolin > taxifolin. QSAR analysis suggest that OH groups in 3, 4′, and 5 positions and C2-C3 double bond are necessary for MPO inhibition [55]. Apart from inhibiting MPO, several flavonoids directly scavenge HOCl, forming the chlorinated flavonoid derivatives. For example, quercetin, in presence of HOCl, is transformed into 6-mono and 6,8-dichlorinated derivatives [56]. 

In animal experimental models, tea and tea-derived flavonoids, red wine-derived flavonoids, and isolated quercetin or catechin, significantly inhibit atherosclerotic lesion development in the apo E deficient mouse [56]. Similar results have been obtained with red grape extracts using cholesterol-fed hamsters [57]. Using high cholesterol-fed rabbits, the antiatherogenic effects of the citrus flavonoids, naringin and naringenin have been demonstrated. These effects are mediated, at least in part, by the inhibition of hepatic acyl CoA: cholesterol acyltransferase (ACAT) activity, the decreased cholesterol absorption and expression of VCAM-1 (vascular cell adhesion molecule-1) and MCP-1 (monocyte chemoattractant protein-1) which play important roles in the adhesion of monocytes to the endothelium [58]. In addition, naringin has been shown to lower hypercholesterolemia-induced intercellular adhesion molecule-1 (ICAM-1) expression on endothelial cells of hypercholesterolemic rabbits [59]. Furthermore, quercetin, which is present in green and black tea, and the black tea flavonoid theaflavin, inhibit the development of atherosclerosis in apo E deficient mice [60,61]. 

### 2.4. Flavonoids and Atherosclerosis: Clinical Studies

Daily administration of 100 mg of (−)-epicatechin and of 160 mg of quercetin-3-glucoside to healthy (pre)hypertensive men and women (40–80 year) produced a decrease of sE-selectin and IL-1β, improving endothelial function [62]. 

The endothelial dysfunction evaluated by reactive hyperemia improved in smokers was reduced by administration of 400 mL green tea [63]. Furthermore, daily consumption of 8 g of green tea inhibits endothelial dysfunction evaluated by flow-mediated forearm dilatation in smokers [64,65]. 

## 3. Flavonoids and Hypercholesterolemia

Cholesterol is transported through the aqueous environment of bloodstream by four different proteins: chylomicron (CM), very low-density lipoprotein (VLDL), low-density lipoprotein (LDL), and high-density lipoprotein (HDL). Chylomicron is formed in the intestinal lymphatic system, and carries out the transport of cholesterol and triacylglycerols (TG) from the intestine to adipose tissue and to skeletal muscles [66]. VLDL, transporting newly synthesized TG and cholesterol from the liver to adipose tissue and skeletal muscles, is formed in liver, whereas LDL, which represents the principal cholesterol carrier in blood, and transport cholesterol to the tissues that need it, is formed in plasma and derives from intermediate-density lipoprotein (IDL) which receives cholesteryl ester (CE) from HDL [67]. HDL takes the excess cholesterol from peripheral tissue and carries it back to the liver, so it is involved in preserving plasma cholesterol homeostasis [67]. Two proteins, i.e., cholesteryl ester transport protein (CETP) and lecithincholesterol acyltransferase (LCAT), are involved in regulating levels of circulating lipoprotein-cholesterol complexes (LDL-c and HDL-c). A further role is played by two receptors, LDL receptor (LDL-R) and scavenger receptor B class 1 (SR-B1) [68]. Lecithin cholesterol acyltransferase is a 416 residues glycoprotein, mainly formed in liver and secreted into plasma where it circulates in association with HDL. The enzyme catalyzes the transfer of the sn-2 fatty acid of lecithin to the free 3-OH group of cholesterol, producing cholesteryl ester and lysolecithin. The main substrate for LCAT is represented by HDL-cholesterol: LCAT plays a central role in the maturation of HDL, and is involved in the determination of HDL composition, structure, intravascular metabolism, and plasma concentration [69]. Low levels of LCAT are associated with low levels of HDL-c [70]. CTEP (cholesteryl ester transfer protein) is involved in lipid metabolism: it aids the exchange of lipids between lipoproteins through the transfer of cholesteryl esters from HDL to LDL or VLDL, lowering HDL levels [71]. LDL receptors are membrane proteins carried to the plasma membrane via the endoplasmic reticulum-Golgi pathway and then taken up via endocytosis followed by recycling or degradation [72]. These receptors bind LDL particles and apolipoprotein B (Apo-B) that are present in the extracellular fluid. The resulting receptor–ligand complex, which is internalized through endocytosis via clathrin-coated pits through interactions involving the LDL receptor adaptor protein, LDLRAP1 (also known as ARH), is carried by early endosomes to the late endosomal compartment, where the acidic environment determines the dissociation of the receptor-ligand complex: while the receptor is recycled to the cell surface, the LDL particles are degraded by lysosomes. High free cholesterol content deriving from hydrolysis of cholesteryl esters present in LDL core, leads to the inactivation of the Sterol Regulatory Element Binding Protein 2 (SREBP-2), which regulates the expression of genes for enzymes that determine the cholesterol synthesis and the LDL receptor [73,74]. While LDL receptor is responsible for the removal of LDL-C from circulation, the HDL receptor SR-B1 exerts the transport of HDL CE to the liver and steroidogenic organs. The upregulation of SR-B1 receptors expression results in a lowering effect versus circulating HDL [75]. Three transcriptional factors contribute to the control of cholesterol metabolism: sterol regulatory element binding protein-2 (SREBP-2), liver X receptor (LXR), and farnesoid X receptor (FXR) [76]. SREBP-2 controls the transcription for LDL receptors, and 3-hydroxy-3-methylglutaryl-CoA (HMG-CoA) reductase; LXR is involved in regulation of the transcription CYP7A1 encoding cholesterol 7R-hydroxylase, and plays a central role in bile acid synthesis; FXR is a bile acid receptor involved in regulation of bile acid synthesis. 

Finally, there are the acyl CoA: cholesterol acyltransferases ACAT-1 and ACAT-2, which catalyse the intracellular esterification of cholesterol. ACAT-2 is required for cholesterol absorption in small intestine, before cholesterol incorporation into CM. The same enzyme in liver has a central role in VLDL formation [76,77]. ACAT inhibition lowers plasma cholesterol level through inhibition of cholesterol absorption in intestine and of VLDL production in the liver. 

### 3.1. Flavonoids and Hypercholesterolemia: Effects on HMGCoA Reductase and LDL Receptors Expression

Oral administration of the quercetin glycoside rutin to streptozotocin-induced diabetic rats has been shown to be able to reduce the levels of lipids in plasma and tissues. In particular, it was observed that rutin increases plasma HDL cholesterol and decreases LDL and VLDL cholesterol: these events are, in part, due to an observed reduction in activity of 3-hydroxy 3-methylglutaryl coenzyme A (HMGCoA) reductase, and to the incease of plasma LPL and LCAT activities [78]. In accordance with these results, administration of *Scutellaria baicalensis* stem-leaf total flavonoid to hyperlipidemic rats, reduces total cholesterol, triglycerides, LDL-C, Apo-B concentration and increases HDL-c; partially due to the observed increased activity of LCAT [79]. 

Isoflavones, flavones, and flavanones reduce blood cholesterol levels through inhibition of cholesterol synthesis and increase of LDL receptor expression [79,80]. Soya isoflavones also affect plasma cholesterol levels [81] through stimulation of the LDL receptor. Dietary isoflavones, such as genistein or daidzein, induce a decrease in plasma cholesterol in C57BL/6 mice but not in LDL receptor-deficient mice [82]. Isoflavonoids such as formononetin, biochanin A, and daidzein increase LDL receptor activity in HepG2 cells [83]. This biological action is probably due to the effect of flavonoids on SREBP-2 [84,85,86,87,88,89,90,91,92,93,94,95]. 

Two isoflavones isolated from bergamot (*Citrus bergamia*) juice, brutieridin and melitidin [84], as well as kaempferol, naringenin, myricetin and EGCG [85] inhibit HMGCoA reductase activity. EGCG competitively binds to the cofactor site of the reductase due to the steric hindrance to both NADPH and HMGCoA binding. Green tea catechins reduce plasma cholesterol in different animal models and influence favourably cholesterol metabolism in cell cultures [96], via the upregulation of LDL receptors occurring through SREBP-2 activation at least in part due (−)-epigallocatechin-gallate (EGCG) [97,98,99,100]. Feeding rats a diet with 2% of green tea catechins results in a significant increase in LDL receptor binding activity [101]. The same effect was observed in rabbits fed a hypercholesterolemic diet, treated with a Green Tea Extract (GTE) [102]. The hypocholesterolemic activity of GTE is associated to the an increased faecal bile acid and cholesterol excretion: in fact GTE administration to hamsters fed a 0.1% cholesterol diet determines a decrease of total cholesterol and triglycerides and an increase in excretion of both neutral and acidic sterols [93]. An analogue effect occurred in rats fed tea extracts [103,104,105,106,107,108].

### 3.2. Flavonoids and Hypercholesterolemia: Effects on ACAT Proteins

Several flavonoids, such as hesperidin, hesperetin, naringin, and naringenin, improve cholesterol metabolism in vivo [97,98]. Naringenin and hesperetin inhibit the accumulation of Apo-B in HepG2 cells, in a concentration-dependent manner. This effect is achieved through inhibition of ACAT-1 and ACAT-2 activities, a selective decrease in ACAT-2 expression, and an inhibition of MTP (microsomal triglyceride transfer protein) activity which results in a lower availability of lipids, in particular CE, essential for Apo-B-containing lipoproteins formation. The increased LDL receptor activity, may be related to the estrogenic activity of these flavonoids, which is involved in the reduction of Apo-B accumulation. In association with these biological events, inhibition of hepatic HMGCoA reductase and ACAT activities and an increase in the faecal acidic sterols have been observed [100]. The administration of hesperetin or hesperetin metabolites, 3,4-dihydroxyphenylpropionic acid (DHPP) (0.012%) and 3-methoxy-4-hydroxycinnamic acid (ferulic acid) (0.013%) to hypercholesterolemic hamsters, , for 12 weeks, results in a significant decrease of plasma total cholesterol, non-high-density lipoprotein-cholesterol (HDL-c), Apo-B, hepatic lipids, and cholesterol regulating enzymes, compared to the control. Ferulic acid has been shown to be more potent in raising HDL-c/total cholesterol ratio [101].

Two flavonoids extracted from *Psoralea corylifolia*, named bavachin and isobavacalchone, and an isoprenyl flavonoid extract from licorice (*Glycyrrhiza glabra*) roots, glabrol, have been shown to inhibit ACAT activity in a concentration-dependent manner and in a non-competitive mode, and thus affect cholesteryl ester formation in human HepG2 cells [109,110]. Naringin and naringenin exert their hypocholesterolemic effect in high cholesterol diet fed rats, in part, through a slight inhibition of hepatic ACAT, even if other mechanisms, such as HMG-CoA reductase inhibition, may be involved.

The effects of rutin and its metabolite, quercetin, on cholesterol metabolism have also been investigated: oral administration of rutin to rats fed a high cholesterol diet results in a decrease in serum level of total and LDL cholesterol and in a decrease of liver enzymes and weight [99]. According to this data, it has been reported [111] that oral administration of rutin to streptozotocin-induced diabetic rats increases HDL cholesterol and reduces levels of LDL- and VLDL-cholesterol, together with the induced decrease of HMGCoA activity and the increase of plasma LPL and LCAT. Since oral administration of rutin to normal rats does not alter these parameters, its beneficial effects may be due to its antioxidant activities. 

### 3.3. Flavonoids and Cholesterol: Clinical Studies

Consumption of soy phytoestrogens results in a decrease of plasma total and LDL- cholesterol in hypercholesterolemic subjects [86,95]. Some authors reported that soy proteins with a high isoflavone content consumption by normocholesterolemic subjects results in a great reduction of LDL-c, in comparison with the same soy intake with a low isoflavone intake [82,83]. Conflicting data have been obtained in two clinical trials where a high isoflavone diet gave the same results, in terms of blood cholesterol, of a low-isoflavone diet [86,87,88,89,90,91,92]. Metanalysis showed that consumption of soy protein with a high isoflavone content results in a stronger hypocholesterolemic activity than the same diet with a low isoflavone content [93]. In another metanalysis [94], which included 23 randomized control trials, it was observed that an isoflavone-rich diet results a great decrease in plasma total cholesterol, LDL-c, triglycerides and a concommittant increase in HDL-c, while tablets based on extracted isoflavones showed no effect on these parameters [95]. 

Furthermore, the administration of bergamot (*C. bergamia* Risso & Poiteau) fruits extract rich in neoeriocitrin, naringin, neohesperidin, melitidin and brutieridin, for 30 days, to patients suffering from hypercholesterolemia, resulted in a dose-dependent reduction of total and LDL cholesterol levels, of triglyceride levels and an increase of HDL-c [106]. 

In addition, antocyanins may be clinically relevant, as supplementation with delphinidin-rich maqui berry extract Delphinol^®^, at the daily dose of 180 mg, to prediabetic individuals, for 3 months, resulted in a decrease of LDL-c [107]. 

Epidemiological data show a negative correlation between tea consumption and plasma levels of total cholesterol and triglycerides in Japanese and Norwegian people. A theaflavin-rich tea extract (375 mg/day) significantly decreases total cholesterol and triglycerides in subjects with mild to moderate hypercholesterolemia [103]. However some conflicting results have been obtained: a cross-sectional study showed no effect of green tea consumption toward lipid levels [104]. This result is likely due to the daily low dose of catechins administered in the study; in fact the administration of a Green Tea Extract (GTE) to postmenopausal women, at the daily dose of 5260 mg of catechins (1932 mg of EGCG), for 12 months, produced a decrease of circulating TC and LDL-cholesterol concentrations, in particular in those with elevated baseline TC concentrations [108].

## 4. Flavonoids and Diabetes: A Snapshot

A direct relationship between hyperglycaemia level and cardiovascular disease morbidity and mortality has been demonstrated. In fact, patients suffering from diabetes and CVD show coronary artery disease, peripheral vascular disease, cerebrovascular disease, diabetic cardiomyopathy, and hypertensive cardiomyopathy [112,113,114,115,116,117,118,119]. This disease represents an increasing public health problem in many countries [120]. Two types of diabetes are discerned: type 1, or insulin-dependent diabetes, where body cannot produce insulin, generally occurs in children and young adults; type 2, noninsulin-dependent, diabetes mellitus, characterized by fasting and postprandial hyperglycaemia and relative insulin insufficiency [120]. In recent years particular attention has been given to the research of hypoglycaemic agents from natural products, in particular from those derived from plants. 

Many studies reveal the potential role of flavonoids in the treatment of diabetes and indicate the hypoglycaemic actions of flavonoids in different experimental models and treatments [121,122,123,124,125]. 

Epigallocatechin gallate, given intraperitoneally to rats, determines a reduction of blood glucose and insulin levels [126,127,128]. The data demonstrate that green tea improves glucose metabolism in healthy humans in oral glucose tolerance tests, and produces an anti-hyperglycaemic effect without affecting insulin secretion in streptozotocin-induced diabetic mice [122]. Also genistein reduces blood glucose levels in diabetic rats, compared with the control, in glucose tolerance tests. Similar data have been obtained with chronic treatments with genistein and daidzein in db/db mice and streptozotocin-induced rats [126,127,128]. 

Some flavonols, such as kaempferol, myricetin, rutin and its metabolite quercetin, show hypoglycemic activity [129,130,131]. In particular, oral administration of rutin to diabetic rats results in a plasma glucose levels reduction [130]. Different studies show that some flavonoids compete with glucose in several absorption mechanisms indicating that intestinal absorption reduction may represent one hypoglycemic effect. In fact, this action was observed into intestinal brush border membrane vesicles of rabbits with a soybean extract which contains the two isoflavones genistein and daidzein [131]. 

### 4.1. Flavonoids and Diabetes: Effects on α-glucosidase 

Another way leading to a reduction of glucose absorption is represented by α-glucosidase inhibition. As regards this biological activity, the two anthocyanins cyanidin-3-α-*O*-rhamnoside and pelargonidin-3-α-*O*-rhamnoside reduce glucose absorption and inhibit α-glucosidase activity in vitro [132]. The latter action was observed also with luteolin, kaempferol, chrysin and galangin [133]. Luteolin-7-glucoside, luteolin, amentoflavone and daidzein are the strongest inhibitors of α-glucosidase [134]. 

### 4.2. Flavonoids and Diabetes: Effects on Kidney Function

To better understand how flavonoids exert their hypoglycaemic activity, their influence towards renal filtration system has been investigated. In particular, a sodium-coupled glucose transporters found on the luminal membrane of the proximal tubule of the kidney, is responsible for the reabsorption of glucose from renal filtrate. Precisely, the presence of glucose in urine is often found in diabetic patients and can lead to severe renal impairment. Different works reported that some flavonoids may interfere with renal glucose reabsorption process. Green tea flavonoids reduce the urinary excretion of proteins and the renal morphological alterations related to diabetic nephropathy and ameliorate blood glucose and glycosylated protein levels [135,136]. Puerarin, another flavonoid, is able to antagonize the increase of collagen IV content in glomerular mesangial cells, so may contribute to the reduction of the aggravating the occurrence and development of diabetic nephropathy [137]. 

### 4.3. Flavonoids and Diabetes: Effects on Pancreas and Insulin Secretion

Insulin exerts a central role in regulating blood glucose. Triggered by glucose, this hormone is secreted into blood circulation by the β-cells of the endocrine portion of pancreas. Glucose enters these cells via a protein named glucose transporter type 2 (GLUT-2). Once inside the cell, glucose gets phosphorylated by glucokinase, the first step of glycolysis, which eventually leads to an increase of ATP, which, binding to ATP-dependent-K^+^-channels, determines the closure of these channels, which in turn causes cells depolarization. This event provokes the activation of voltage-sensitive calcium channels, triggering a calcium influx, followed by insulin secretion [138]. Flavonoids may influence the synthesis and the release of insulin from β-cells. In streptozotocin-diabetic rats, oral administration of genistein has been shown to incease insulin secretion from mouse pancreatic islets, in presence of glucose [139]. The mechanism underlying this biological effect may involve a rise in intracellular cAMP through the increase of adenylate cyclase activity and the activation of protein kinase A (PKA), which suggests that genistein regulates the insulinotropic action through the activation of the cAMP/PKA signalling cascade [140]. In addition, green tea catechins exert a hypoglycemic activity in vitro and in vivo. In fact, chronic administration of a green tea-based supplement results in an increase of the basal and insulin-stimulated glucose uptake in adipocytes [141,142]. 

Several in vitro studies show the protective effects of anthocyanins towards pancreatic β-cells [143,144]. Chinese bayberry anthocyanins exert a protective activity towards pancreatic β-cells INS-1 against induced necrosis and apoptosis, through ERK1/2- and PI3K/Akt-mediated heme oxygenase-1 upregulation. 

Anthocyanins and anthocyanidins induce insulin secretion from β-cells. In particular, cyanidin-3-glucoside is less potent than delphinidin-3-glucoside at a lower glucose concentration, while it is more potent at a higher glucose concentration; pelargonidin-3-galactoside does not affect insulin secretion, while cyanidin-3-galactoside does, in the same conditions. These data suggest that the number of hydroxyl groups in ring B of anthocyanins is relevant for their ability to induce insulin secretion [144,145]. 

Cyanidin-3-glucoside (C3G), and cyanidin-3-rutinoside (C3R) induce insulin secretion in MIN 6 cells, through the upregulation of glucokinase and the activation of the GLP-1 receptor, triggering the intracellular ATP accumulation, and favour β-cells survival through the improved expression of duodenal homeobox factor-1 (PDX-1) [145]. Formononetin exerts antidiabetic effects through several mechanisms including the inhibition of islet B cell apoptosis and the induction of islet B cell regeneration down-regulating the Fas and Caspase-3 mRNA and protein levels and up-regulating the PDX-1 and insulin receptor substrate 2 (IRS2) mRNA. Furthermore, it induces insulin secretion, and up-regulates the GK and GLUT2 mRNA and protein levels in pancreas tissue from mice with alloxan-induced type 1 diabetes [146] (Quercetin also stimulates insulin release, but with a different mechanism, involving transient KATP channel inhibition and ICa stimulation [147]). 

### 4.4. Flavonoids and Diabetes: Clinical Studies

A clinical study indicates that oral administration of silymarin results in a reduction of glycosuria and glycaemia, even after four months of treatment [135].

In a randomized, placebo-controlled, double-blind trial, it has been demonstrated that daily administration of 320 mg of anthocyanins, for 24 weeks, to diabetic patients, lowers fasting plasma glucose, and decreases serum levels of LDL cholesterol and triglycerides [148]. One-year treatment with genistein, at a daily dose of 54 mg, of Caucasian postmenopausal women with metabolic syndrome, resulted in a decrease of fasting glucose, fasting insulin, and insulin resistance, total cholesterol, LDL-C, triglycerides, visfatin, homocysteine and an increase of HDL-c and adiponectin [149]. Furthermore, the administration of 27 g/day (split dose) flavonoid-enriched chocolate (containing 850 mg flavan-3-ols [90 mg epicatechin] and 100 mg isoflavones) to patients with type 2 diabetes, for one year, reduced peripheral insulin resistance, improved insulin sensitivity, and led to a decrease in total cholesterol to HDL-c ratio, and a dcrease in LDL-c [150]. 

## 5. Conclusions and Perspectives

Atherosclerosis is considered one of the main medical and social problems in the industrialised world, as it is strongly related to a plethora of chronic pathologies associated to an increase of morbidity and mortality. Atherosclerosis has a multifactorial etiology, and it is associated to degenerative changes in the wall of large arteries, inhibiting or suppressing blood flow to organs and tissues. The excessive accumulation of lipids in the arterial intima represents a key step of the atherosclerotic process. Also, oxidative stress and inflammation contribute to the onset and progression of atherosclerosis. At present, there are no direct anti-atherosclerotic drugs, and the main strategy to prevent this pathology is advice for a healthy lifestyle and, where necessary, prescription of antiperlipidemic, antidiabetic and antihypertensive drugs. 

In this work we aim to focused on naturally occurring flavonoids able to act as multitarget compounds, affecting several molecular networks activities and potentially providing preventive and curative effects in patients with atherosclerosis, or at high risk of atherosclerosis. We recently demonstrated the importance of kaempferol [151] and of kerala powder [152] and how the food tend to influence the wellbeing. However, still a lot of efforts are necessary to better explore the many pathways of both cardiovascular diseases and diabetes.

Flavonoids may be relevant in the prevention and treatment of atherosclerosis and atherosclerosis-related disorders as they act as antioxidant, hypocholesterolemic and antidiabetic agents. Therefore, is promising to investigate different flavonoids metabolites that could help to prevent and/or manage certain type of disorders. The great potential of these compounds should goad to probe deeply the molecular mechanisms involved.
